# MALDI Mass Spectrometry Imaging Reveals Decreased CK5 Levels in Vulvar Squamous Cell Carcinomas Compared to the Precursor Lesion Differentiated Vulvar Intraepithelial Neoplasia

**DOI:** 10.3390/ijms17071088

**Published:** 2016-07-08

**Authors:** Chao Zhang, Georgia Arentz, Lyron Winderbaum, Noor A. Lokman, Manuela Klingler-Hoffmann, Parul Mittal, Christopher Carter, Martin K. Oehler, Peter Hoffmann

**Affiliations:** 1 Adelaide Proteomics Centre, School of Biological Sciences, The University of Adelaide, Adelaide 5005, Australia; chao.zhang01@student.adelaide.edu.au (C.Z.); georgia.arentz@adelaide.edu.au (G.A.); lyron.winderbaum@student.adelaide.edu.au (L.W.); noor.lokman@adelaide.edu.au (N.A.L.); manuela.klinglerhoffmann@adelaide.edu.au (M.K.-H.); parul.mittal@adelaide.edu.au (P.M.); 2 Institute for Photonics and Advanced Sensing (IPAS), The University of Adelaide, Adelaide 5005, Australia; 3 Discipline of Obstetrics and Gynaecology, School of Medicine, Research Centre for Reproductive Health, Robinson Institute, Adelaide 5005, Australia; martin.oehler@adelaide.edu.au; 4 Department of Cytopathology, SA Pathology, Adelaide 5005, Australia; Christopher.Carter@sa.gov.au; 5 Department of Gynaecological Oncology, Royal Adelaide Hospital, Adelaide 5005, Australia

**Keywords:** Matrix-assisted laser desorption/ionization (MALDI), imaging mass spectrometry (MSI), differentiated vulvar intraepithelial neoplasia (dVIN), vulva squamous cell carcinomas (VSCC), Cytokeratin 5 (CK5)

## Abstract

Vulvar cancer is the fourth most common gynecological cancer worldwide. However, limited studies have been completed on the molecular characterization of vulvar squamous cell carcinoma resulting in a poor understanding of the disease initiation and progression. Analysis and early detection of the precursor lesion of HPV-independent vulvar squamous cell carcinoma (VSCC), differentiated vulvar intraepithelial neoplasia (dVIN), is of great importance given dVIN lesions have a high level of malignant potential. Here we present an examination of adjacent normal vulvar epithelium, dVIN, and VSCC from six patients by peptide Matrix-assisted laser desorption/ionization Mass Spectrometry Imaging (MALDI-MSI). The results reveal the differential expression of multiple peptides from the protein cytokeratin 5 (CK5) across the three vulvar tissue types. The difference observed in the relative abundance of CK5 by MALDI-MSI between the healthy epithelium, dVIN, and VSCC was further analyzed by immunohistochemistry (IHC) in tissue from eight VSCC patients. A decrease in CK5 immunostaining was observed in the VSCC compared to the healthy epithelium and dVIN. These results provide an insight into the molecular fingerprint of the vulvar intraepithelial neoplasia that appears to be more closely related to the healthy epithelium than the VSCC.

## 1. Introduction

Vulvar cancer is the fourth most common gynecological cancer worldwide, constituting 5% of all gynecological cancers and 0.6% of all cancer cases in women [1]. In 2015, 5150 new cases were diagnosed in the United States with 1080 recorded deaths [1]. The majority of vulvar cancers are squamous cell carcinomas (VSCC), of which there are two etiological pathways: one linked to human papilloma virus (HPV) infection, and the other HPV-independent with genetic alterations. The HPV related and non-HPV related forms of vulvar cancer have distinct precursor lesions, known as usual-type vulvar intraepithelial neoplasia and differentiated vulvar intraepithelial neoplasia, respectively. Mutations in the tumor suppressor gene TP16 are associated with HPV related VSCC, while mutations in TP53 have been shown to promote the development of non-HPV related VSCC from the inflammatory condition lichen sclerosus [2].

Recently, the incidence rates of non-HPV related invasive vulvar cancer and its precursor lesion, differentiated vulvar intraepithelial neoplasia (dVIN), have significantly increased amongst young women [3,4]. In Australia, the incidence rates of the disease are extremely high among Aboriginal women from Arnhem Land in the Northern Territory. Studies conducted within this cohort have found no relation between the disease and HPV infection or any underlying genomic irregularities that differentiate this group from other VSCC patients [3,4].

For many years the clinicopathological significance of vulvar intraepithelial neoplasia (VIN) as a precursor to vulvar squamous cell carcinoma (VSCC) was questioned [5] due to the low detection rates of dVIN as a solitary lesion [6]. However, in recent times, dVIN has been confirmed as the direct precursor lesion to HPV-independent vulvar carcinoma, which in the past may have been frequently misdiagnosed as lichen sclerosus or other forms of dermatoses [7,8]. Such misdiagnoses were in part due to numerous changes over the last few decades with regards to the terminology and criteria used to differentiate lichen sclerosus from dVIN and other inflammatory conditions such as squamous cell hyperplasia and lichen simplex chronicus [7,9,10].

The detection of dVIN as a solitary lesion is of great importance given the lesions have a high level of malignant potential [6], with studies showing the median time between the diagnoses of dVIN to VSCC development around 23 to 28 months [6,7]. Mutation of TP53 is a known early event in the development of dVIN [11] and the detection of dVIN adjacent to VSCC is high at around 80%. However, the detection of dVIN as a solitary lesion is scarce [6] and can be difficult to diagnose due to the subtle histological appearance of the lesion [12,13]. Currently the characterization of the dVIN at the molecular level has been poor due to sample rarity, hence the understanding of VSCC progression from healthy tissue to the precursor lesion is low compared to other well studied carcinomas.

MALDI-MSI allows the in situ analysis of tissue sections combining classical mass spectrometry with histological tissue analysis [14]. MALDI-MSI can identify peptides and other molecules from samples whilst retaining the important spatial information of the tissue. Previous studies in the gastrointestinal tract, lung, brain and other gynaecological cancers have shown that MALDI-MSI can acquire a more comprehensive proteomic picture than immunohistochemical testing [14,15,16,17]. Here we present an examination of adjacent normal vulvar epithelium, dVIN, and VSCC from six patients by peptide MALDI-MSI. All patients included in this study were HPV negative and lichen sclerosus positive. The results revealed the differential detection of multiple peptides from the protein cytokeratin 5 (CK5) across the three tissue types. The difference observed in the relative abundance of CK5 by MALDI-MSI between the healthy epithelium, dVIN, and VSCC was further investigated by IHC in eight tissues from VSCC patients. This revealed a decrease in CK5 immunostaining in the VSCC as compared to the healthy epithelium and dVIN.

## 2. Results

### 2.1. Matrix-Assisted Laser Desorption/Ionization Mass Spectrometry Imaging (MALDI-MSI)

MALDI-MSI was carried out on tissue sections containing regions of healthy vulvar epithelium, dVIN, and VSCC from six late stage carcinoma patients. Peak groups, representing the imaged peptides across all of the analyzed tissue sections with the same *m*/*z* (assuming a mass accuracy of ±0.02 Da), were detected using density-based clustering of peaks (DBSCAN* [18] with an epsilon of 0.02 Da and a minimum of 100 points) and the stringent criterion of requiring peak groups to contain a minimum of 10,000 peaks. This stringent criterion of a minimum of 10,000 peaks per peak group was initially put in place to ensure that the peak groups analyzed could be reproducibly detected across a majority of spectra. With this criteria in place, 31 peak groups were detected (Table 1). When the minimum number of peaks per peak group was lowered to 1000, 316 peak groups were detected. For each peak group the abundance weighted mean (AWM) was calculated, representing the apex of the peak group and the *m*/*z* of the most intense peptide within the group. The widths of the 31 detected peak groups ranged from ±0.075 to ±0.9 Da. The maximum difference, d, in median log intensity between the three tissue regions was calculated for each of the 31 peak groups of interest across the patient cohort (Table 1). Of the 31 peak groups, 19 were found to have a difference in fold change intensity of ≥1.4-fold across the three tissue types.

### 2.2. Cytokeratin 5 (CK5) Identified as a Protein of Interest

**Figure 1:**
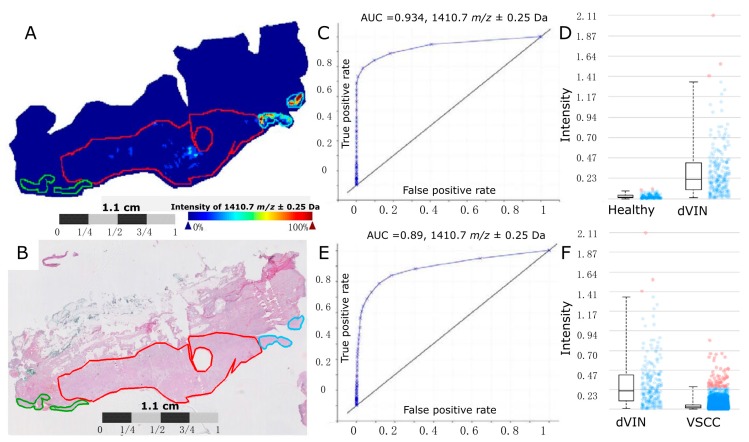
A MALDI-MSI annotated ion intensity map from a representative vulvar tissue section for the peak group *m*/*z* 1410.67. (**A**) An ion intensity map for *m*/*z* 1410.67, which matches to the CK5 peptides SFSTASAITPSVSR and TTAENEFVMLKK (both with a [M + H] *m*/*z* 1410.67). The regions of healthy epithelium are outlined in green, the dVIN is outlined in blue, and the VSCC is outlined in red; (**B**) H & E stain of Healthy (green), dVIN (blue) and VSCC (red) tissue post MALDI-MSI data acquisition; (**C**) A ROC curve and (**D**) boxplot analysis of the MALDI-MSI data for *m*/*z* 1410.67 comparing the annotated healthy epithelium to the dVIN region, AUC = 0.934; (**E**) A ROC curve and (**F**) boxplot analysis of the MALDI-MSI data for *m/z* 1410.67 comparing the annotated dVIN to the VSCC region, AUC = 0.89. Blue dots represent the *m/z* 1410.67 spectra intensity interval between the lower and upper quartiles. Red dots represent the spectra intensities outside of the intervals.

In order to gain peptide identifications for the MALDI-MSI peak groups of interest nanoflow liquid chromatography tandem mass spectrometry (nano-LC-MS/MS) was performed on laser microdissected regions of the healthy epithelium, dVIN, and VSCC. Matching between the MALDI-MSI peak groups and nano-LC-MS/MS data was done by aligning the experimental *m*/*z* values of the sequenced peptides that fell between the minimum and maximum *m*/*z* of each of the MALDI-MSI peak groups of interest. A table containing all of the matching results for the 31 peak groups of interest is provided in Table S2. Of the 31 MALDI-MSI peak groups of interest, six matched to sequenced peptides from Cytokeratin 5 (CK5) (Table 2), hence CK5 was selected for further analysis. One of the peak groups (AWM [M + H] of 1410.72) matched to two unique CK5 peptides that share the same mass to within 0.009 Da, SFSTASAITPSVSR (1409.7203) and TTAENEFVMLKK (1409.7295). A MALDI-MSI annotated ion intensity map for the peak group *m*/*z* 1410.67, which matches to the CK5 peptides SFSTASAITPSVSR and TTAENEFVMLKK (both with a *m*/*z* 1410.67), is shown in Figure 1. With regards to the remaining results in Table 1, only three other proteins were detected with more than one unique peptide match as shown in Table S2. Four peptides were detected from the protein Neuroblast differentiation-associated protein AHNAK, three peptides were detected from the protein Annexin A4, and two peptides were detected from the protein Nicotinate phosphoribosyltransferase.

### 2.3. Immunohistochemistry (IHC) Analysis of CK5 across the Healthy Epithelium, Differentiated Vulvar Intraepithelial Neoplasia and Vulvar Squamous Cell Carcinoma

**Figure 2:**
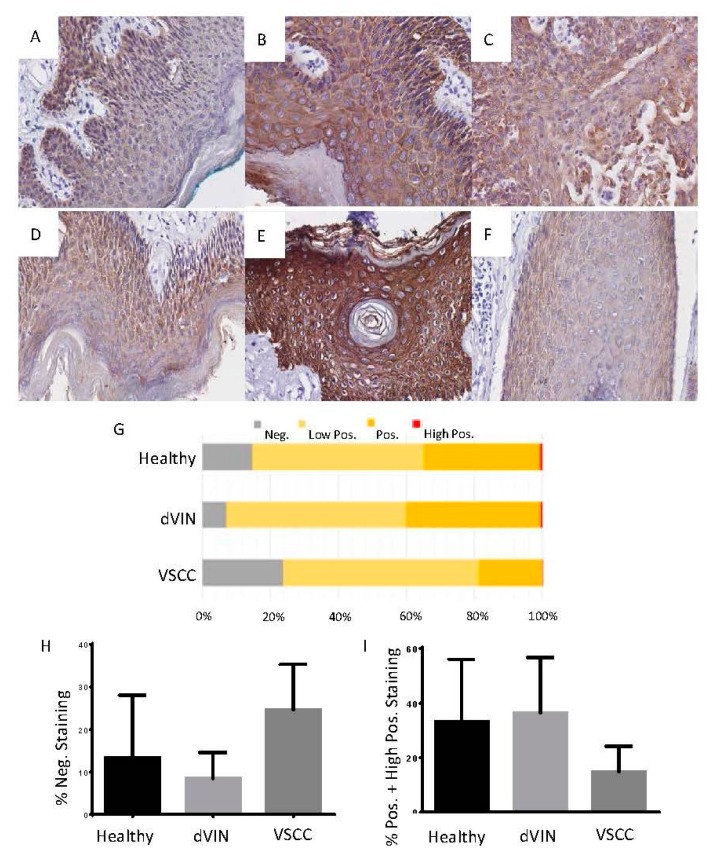
IHC analysis of CK5 on vulvar tissues containing regions of healthy epithelium, dVIN, and VSCC, all at 40× magnification. (**A**) CK5 staining of healthy epithelium; (**B**) dVIN; (**C**) VSCC from a single section; (**D**) CK5 staining of healthy epithelium; (**E**) dVIN; (**F**) VSCC from a single section of a different patient; (**G**) Quantitative analysis of CK5 staining across the tissue types was performed using IHC profiler-Image J. For each tissue section, three representative photo-micrographic images at 40× magnification were used and each image was assigned a staining score of negative, low positive, positive, and high positive; (**H**) The percent of negative staining was significantly different between the dVIN and the VSCC (*p* < 0.0001), but not between the dVIN and healthy epithelium (*p* = 0.11); (**I**) The percent of positive and high positive staining was significantly different between the dVIN and the VSCC (*p*< 0.0001), but not between the dVIN and healthy epithelium (*p* = 0.59).

**Figure 3:**
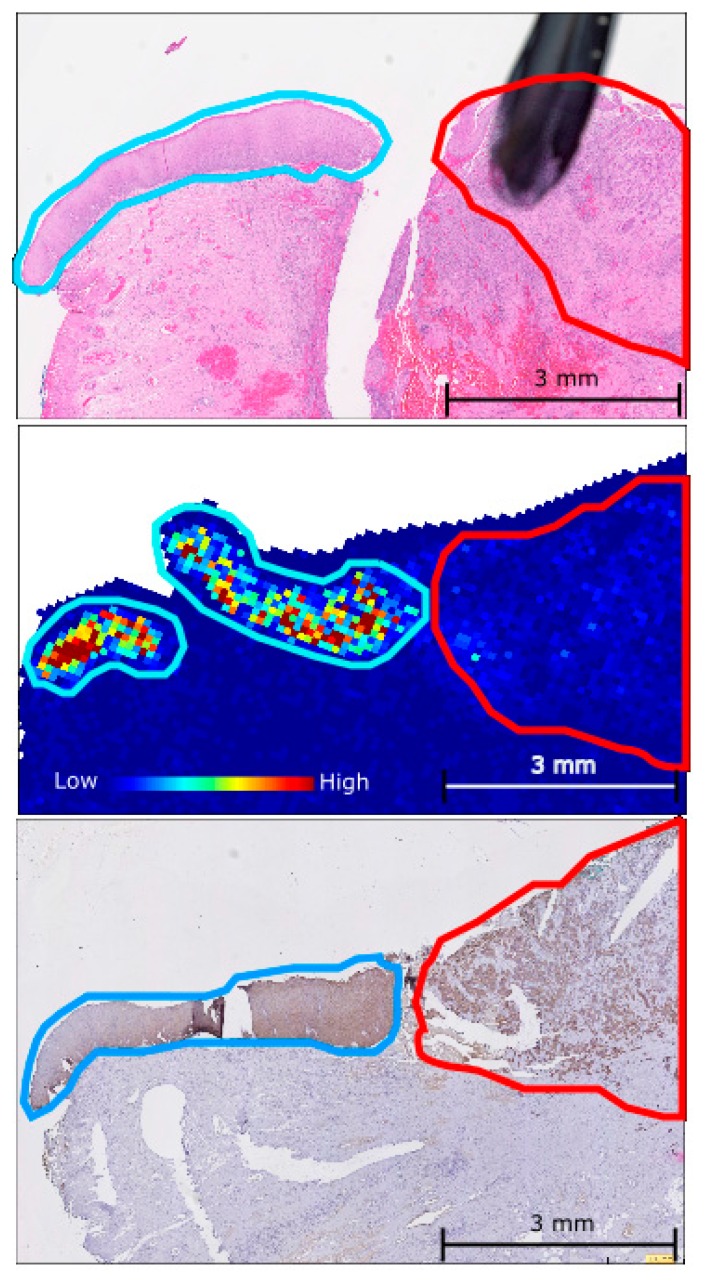
A comparison of the expression pattern observed for the CK5 peptides SFSTASAITPSVSR and TTAENEFVMLKK, both *m*/*z* 1410.72, in the dVIN (blue) and VSCC (red) using: MALDI-MSI ion intensity map (**middle**); H & E staining (**top**); and CK5 IHC staining (**bottom**) on consecutively cut vulvar tissue sections.

The differential detection of CK5 across the three tissue types as determined by MALDI-MSI was further investigated by IHC in tissue from eight VSCC patients. Of these eight tissues, three had been previously analyzed in the MALDI-MSI experiments. IHC analysis of CK5 revealed a significant difference in the level of staining between the dVIN (mean 91.6 ± 6.1) and VSCC (mean 75.4 ± 10.7) tissues (*p* = 1.758 × 10^−7^). A similar result was observed for the healthy as compared to VSCC tissue (*p* = 0.004), with increased staining observed in the healthy epithelium (mean 86.7 ± 14.8). Overall, there was no significant difference in the level of staining between the dVIN and healthy epithelium (*p* = 0.14), although the mean staining intensity in the dVIN was slightly higher. Results for the IHC analysis and CK5 staining from two representative vulvar tissues are provided in Figure 2. A direct comparison of the CK5 peptides SFSTASAITPSVSR and TTAENEFVMLKK, both *m*/*z* 1410.72, as detected by MALDI-MSI to the detection of CK5 by IHC on consecutive vulvar tissue sections is shown in Figure 3.

## 3. Discussion

Due to the relative rarity of vulvar cancer, characterization of the disease at the molecular level has been poor. A greater understanding of vulvar cancer progression is required, however, especially with regards to dVIN as the precursor lesion is known to have a high level of malignant potential [6] and is difficult to diagnose [12,13]. Generally, dVIN is characterized by thickened parakeratotic epithelium with elongation and anastomosing rete ridges, little to no atypia above the basal or parabasal layers of the epidermis, with the basal cell layer containing abnormal squamous cells undergoing atypical mitosis and high levels of epithelial cell differentiation in the upper epithelium [13,19,20]. The aim of this study was to in situ characterize the dVIN lesions at the peptide level using MALDI-MSI for comparison to healthy vulvar epithelium and VSCC. To the best of our knowledge, this is the first report performing such an analysis from FFPE vulvar cancer tissues.

MALDI-MSI was carried out on tissue sections containing regions of healthy vulvar epithelium, dVIN, and VSCC from six late stage carcinoma patients. Thirty-one peak groups, representing the in situ imaged peptides, could be reproducibly detected across the majority of acquired spectra (Table 1). The median log intensity for each of the 31 peak groups was calculated for the regions of healthy epithelium, dVIN, and VSCC, and the maximum difference in intensity between the tissue types was calculated. This revealed 19 of the peak groups to have a change in intensity of ≥1.4-fold across the three tissue types, of which six matched to unique peptides from the protein CK5 (Table 2).

CK5 is a 58 kDa intermediate filament protein that dimerizes with cytokeratin 14 to form the cytoskeleton of basal epithelial cells [21]. CK5 is known to have high levels of expression in squamous cell carcinomas and can be used as a marker of differentiation, with weaker to no expression in adenocarcinomas [22]. Given squamous cell carcinomas account for over 90% of all vulvar cancers and that CK5 is a structural protein of basal epithelial cells [23], the detection of CK5 by both MALDI-MSI was to be expected. A study characterizing the expression of CK5 in serous gynecological carcinomas of the ovaries, endometrium, fallopian tube, primary peritoneum, and cervix observed the highest level of CK5 expression in the cervical and ovarian serous carcinomas, with little to no reactivity in the endometrial, fallopian tube, or primary peritoneum serous carcinomas [24].

In order to determine if there was a genuine difference in CK5 levels across the three tissue types, IHC staining was performed on vulvar tissue from eight VSCC patients. Of these eight tissues, three had been previously analyzed in the MALDI-MSI experiments, six were moderately differentiated, one was well differentiated, and one was poorly differentiated. A degree of keratinization was reported for all of the VSCC with the exception of the poorly differentiated tumor and one of the moderately differentiated tumors. Generally, the expression of CK5 appeared to be diffuse and cytosolic across the three tissue types, with no distinct tissue specific staining patterns observed. IHC analysis revealed a significant increase in the level of CK5 staining in the dVIN as compared to the VSCC legions, and in the healthy epithelium as compared to the VSCC legions (Figure 2). There was a slight increase in the level of CK5 staining detected in the dVIN as compared to the healthy epithelium, but the difference was not significant.

To verify the spatial expression pattern of the peptides detected by MALDI-MSI, consecutive tissue sections that had been H&E stained and analysed by CK5 IHC were compared to ion intensity maps generated for the CK5 peptides from the MSI experiments. A representative example of H&E staining, CK5 IHC, and an ion intensity map for the CK5 peptides SFSTASAITPSVSR and TTAENEFVMLKK, both *m*/*z* 1410.72, from consecutive vulvar tissue sections is shown in Figure 3. The intensity and spatial distribution of the CK5 peptides as detected by MALDI-MSI corroborated well with the CK5 immunostaining observed. These results demonstrate the power of MALDI-MSI in the ability to combine classical mass spectrometry with histological tissue analysis.

This study provides an insight into the molecular fingerprint of the VSCC precursor lesion dVIN, which appears to more closely resemble healthy epithelium than is does cancerous tissue, even in later stage patients. To our knowledge, this is the first MALDI imaging mass spectrometry analysis of vulva cancer.

## 4. Materials and Methods

### 4.1. Sample Cohort

Formalin-fixed paraffin-embedded (FFPE) samples diagnosed with advanced VSCC from 2001 to 2013 were retrieved from the archive of SA Pathology at the Royal Adelaide Hospital (RAH). Ethics approval for the study was granted by the RAH. The age of patients ranged from 37 to 83 years with median age of 66.5 years, all patients were HPV negative, and each tissue analyzed contained regions of healthy vulvar epithelium, dVIN, and invasive vulvar squamous carcinoma (VSCC). Nine of the vulvar carcinomas analyzed were stage III and three were stage IB. Clinical information for the patients is provided in Table S1. All tissues analyzed in this study were annotated by a pathologist.

### 4.2. MALDI-MSI Preparation and Acquisition

MALDI-MSI of FFPE tissue sections was performed on tissue from 6 VSCC patients as previously described [25]. Briefly, tissues were sectioned 8 μm thick and mounted on the Indium-Tin-Oxide (ITO) coated glass slides (Bruker Daltonics, Bremen, Germany) by heating at 60 °C for 1 h. Tissues were deparaffinised in xylene for 5 min, following by two 2 min incubations in 100% ethanol, and two 5 min incubations in 100 mM NH_4_HCO_3_. The tissues were then subjected to heat induced citric acid antigen retrieval (CAAR) (10 mM citric acid, pH = 6) [26] followed by digestion with trypsin gold (Promega, Madison, WI, USA) using an ImagePrep station (Bruker Daltonics, Bremen, Germany) at 37 °C for 2 h. Peptide Internal calibrants (Angiotensin I, [Glu1]-Fibrinopeptide B, Dynorphin A and ACTH fragment [1,2,3,4,5,6,7,8,9,10,11,12,13,14,15,16,17,18,19,20,21,22,23,24]) and α-cyano-4-hydroxycinnamic acid (CHCA) matrix was overlaid onto the tissue sections using an ImagePrep station (Bruker Daltonics, Bremen, Germany) [27]. An ultrafleXtreme MALDI-TOF/TOF MS system (Bruker Daltonics, Bremen, Germany) was used for data acquisition in positive reflectron mode as monitored by flexControl (V3.0.1 Bruker Daltonics, Bremen, Germany) using following settings: 2 kHz, *m*/*z* 800–4000, with 100 μm spatial resolution. Following data acquisition slides were Heamatoxylin and Eosin (H & E) stained and scanned using a NanoZommer (Hamamatsu, Japan). The H & E stained images were then co-registered with the collected MALDI MSI spectra using the flexImaging software (V4.0.1 Bruker Daltonics, Bremen, Germany).

### 4.3. MALDI-MSI Data Analysis

Acquired spectra were recalibrated using the internal calibrants and pre-processed using flexAnalysis and flexImaging software (V4.0.1 Bruker Daltonics, Bremen, Germany) in order to produce peak lists. During the pre-processing the Snap Algorithm with a signal to noise ratio of 2 was used for peak detection, a baseline subtraction was performed using the TopHat method, and baseline smoothing was performed using the Gauss algorithm. Tissue specific regions of interest (ROI) (i.e., healthy epithelium, dVIN and VSCC) were selected and exported into flexImaging (Bruker, Germany). Peaklists from all ROI were combined and density based clustering (DBSCAN* [18], with an epsilon of 0.02 and minimum density of 100 peaks) was used to cluster peaks into peak groups based on their *m*/*z* values. Initially only peak groups containing at least 10,000 peaks were considered for further analysis. This strict criterion was put in place to ensure that all peak groups analyzed could be reproducibly detected in many spectra. The median log intensity in each of the 3 tissue types was calculated for each peak group and for each patient. Peak groups were heuristically ranked by the largest difference in median log intensity between tissue types. The raw data was also analyzed using the SCiLS lab software (SCiLS, GmbH, Bremen, Germany, 2015b) where the processing steps of baseline removal and normalization were carried out [28]. Ion intensity maps for the peptides of interest were generated using the SCiLS lab software.

### 4.4. Peptide Identification by Nanoflow Liquid Chromatography Tandem Mass Spectrometry (nano-LC-MS/MS) 

Tissue areas of healthy, dVIN, and VSCC were collected using laser capture microdissection (LCM) for protein digestion and identification by nano-LC-MS/MS. The FFPE tissue was sectioned at 8 μm thickness, water bath mounted onto PEN membrane slides (Micro-Dissect, Herborn, Germany), and deparaffinised as described above. Areas of healthy epithelium, dVIN, and VSCC were dissected using a Leica AS LCM microscope (Leica Microsystems, Wetzlar, Germany) into 20 μL of 10 mM citric acid buffer (pH = 6) and subjected to heat induced antigen retrieval as described above. Samples were buffered with 10 mM of NH_4_HCO_3_ and digested with 100 ng of trypsin gold (Promega, Madison, WI, USA) overnight at 37 °C.

Nano-LC-MS/MS was performed using an Ultimate 3000 RSLC system (Thermo-Fisher Scientific, MA, USA) coupled to an Impact II™ QTOF mass spectrometer (Bruker Daltonics, Billerica, MA, USA) via an Advance CaptiveSpray source (Bruker Daltonics). Peptide samples were pre-concentrated onto a C18 trapping column (Acclaim PepMap100 C18 75 μm × 20 mm, Thermo-Fisher Scientific) at a flow rate of 5 μL/min in 2% ACN 0.1% TFA for 10 min. Peptide separation was performed using a 75 μm ID C18 column (Acclaim PepMap100 C18 75 μm × 50 cm, Thermo-Fisher Scientific) at a flow rate of 0.2 μL/min using a linear gradient from 5% to 45% B (A: 5% ACN 0.1% FA, B: 80% ACN 0.1% FA) over 130 min, followed by a 20 min wash with 90% B, and a 20 min equilibration with 5% A. MS scans were acquired in the mass range of 300 to 2200 *m*/*z* in a data-dependent fashion using Bruker’s Shotgun Instant Expertise™ method. Singly charged precursor ions were excluded from acquisition. Collision energy ranged from 23% to 65% as determined by the *m*/*z* of the precursor ion.

Acquired spectra were subjected to peak detection, de-convolution, and re-calibration according to a lock mass using DataAnalysis (Version 4.2, Bruker Daltonics). Processed spectra were then exported to Mascot generic format and submitted to Mascot (Version 2.3.02) for identification. Search parameters were as follows; SwissProt Homo sapiens database was searched, the digestion enzyme was specified as trypsin with up to 2 missed cleavages, variable modification of oxidation of methionine, MS mass tolerance of 40 ppm, and MS/MS mass tolerance of 0.2 Da. In Mascot, the peptide false discovery rate was set to <1% using Percolator, the peptide ion score cut off was set to 20, and the peptide significance score was set to <0.05.

### 4.5. Matching the MALDI-MSI Peak Groups to the Nanoflow Liquid Chromatography Tandem Mass Spectrometry (Nano-LC-MS/MS) Results

Matching between the two data sets was completed manually by comparing the experimental *m*/*z* values of the nano-LC-MS/MS sequenced peptides that fell between the minimum and maximum *m*/*z* of each of the MALDI-MSI peak groups.

### 4.6. Immunohistochemistry

For the analysis of Cytokeratin 5 by IHC, tissue from 8 VSCC patients was analyzed as previously described [29]. Briefly, 8 µm tissue sections were placed on plain glass slides, de-waxed, rehydrated and subjected to microwave antigen retrieval in 10 mM citric acid buffer (pH = 6) for 10 min at 100 °C in a steam microwave (Sixth Sense, Whirlpool, VIC, Australia). Tissue sections were then incubated overnight at 4 °C with the Cytokeratin 5 antibody (rabbit monoclonal antibody at a 1/200 dilution, Abcam, Cambridge, MA, USA) in 5% goat serum blocking buffer, followed by incubation with biotinylated anti-rabbit immunoglobulin (1/400, Dako, North Sydney, NSW, Australia) and streptavidin-HRP (1/500, Dako, Australia). Diaminobenzidine (DAB)/H_2_O_2_ (Sigma Aldrich) substrate was added before counterstaining with haematoxylin (Sigma Aldrich), dehydrating and mounting in Pertex (Medite Medizintechnik, Germany). The CK5 stained sections were scanned using a NanoZommer (Hamamatsu, Japan) and viewed in NDP View (Hamamatsu, Japan). For each tissue section, three representative photo-micrographic images from areas of healthy epithelium, dVIN, and VSCC were captured at 40× magnification. Analysis was carried out using the IHC Profiler in ImageJ which determines areas of high positive, positive, low positive, and negative staining [30]. The levels of CK5 positive staining for each tissue type were summed and compared across patients using GraphPad Prism 6 v008 (GraphPad Software, La Jolla, CA, USA), where the mean, standard error of the mean, and significance (paired *t*-test) were calculated.

## Figures and Tables

**Table 1:** Matrix-assisted laser desorption/ionization mass spectrometry imaging peak groups ranked heuristically by the largest difference in median log intensity between the healthy epithelium, differentiated vulvar intraepithelial neoplasia, and vulvar squamous cell carcinoma.

Peptide AWM [M + H] of the Peak Group ^1^	Da Range of the Peak Group	No. Spectra in Peak Group ^2^	Maximum Difference Median Log Intensity ^3^
1198.71	±0.62	23,099	0.75
1028.62	±0.56	15,354	0.66
1410.72 *	±0.82	18,784	0.58
1905.95	±0.52	10,956	0.56
1095.58	±0.82	21,704	0.55
944.54	±0.69	19,354	0.53
957.63	±0.65	10,464	0.52
857.60	±0.68	14,956	0.50
976.51	±0.65	15,534	0.49
878.56	±0.68	11,213	0.46
810.48 *	±0.76	17,095	0.45
958.59	±0.69	15,432	0.42
943.62	±0.82	20,030	0.41
865.42 *	±0.84	15,617	0.41
1217.66	±0.55	10,906	0.39
884.52 *	±0.66	14,337	0.37
856.67	±0.74	15,483	0.36
1143.65 *	±0.64	17,685	0.36
1045.62	±0.65	16,370	0.35
2147.19	±0.14	20,418	0.33
1105.59	±0.50	11,479	0.30
1296.68	±0.10	22,823	0.29
870.64	±0.85	22,749	0.29
842.60	±0.90	25,210	0.22
1267.65	±0.32	11,686	0.20
2932.59	±0.07	15,500	0.19
816.50	±0.73	10,983	0.19
971.56 *	±0.65	15,998	0.18
1111.58	±0.51	13,473	0.09
1570.70	±0.17	12,029	0.05
864.63	±0.70	11,114	0.05

^1^ The abundance weighted means (AWM) *m*/*z* was calculated for the overall peak group; ^2^ Number of Spectra in each peak group compiled from all acquisition spectra across the patient cohort; ^3^ Peak groups were heuristically ranked based on the maximum difference, d, in median log intensity between the tissue regions of interest; * CK5 peptides.

**Table 2:** CK5 matrix-assisted laser desorption/ionization mass spectrometry imaging peak groups of interest with matched nanoflow liquid chromatography tandem mass spectrometry results.

Peptide AWM [M + H] ^1^	*d* ^2^	LC-MS/MS *m*/*z* ^3^	LC-MS/MS MR ^4^	Peptide Score ^5^	Peptide Significance ^6^	Peptide Sequence ^7^
810.48	0.45	405.7087	809.4028	21.44	0.0072	QSSVSFR
865.42	0.41	433.1998	864.3851	26	0.0025	SGGGGGGGFGR
884.52	0.37	442.7286	883.4427	24.74	0.0034	TSFTSVSR
971.56	0.18	486.2429	970.4712	25.1	0.0031	FVSTTSSSR
1143.65	0.36	572.3175	1142.6205	39.59	0.00011	LAELEEALQK
1410.72	0.58	705.8674	1409.7203	115.28	3 × 10^−12^	SFSTASAITPSVSR
1410.72	0.58	705.872	1409.7295	150.55	8.8 × 10^−16^	TTAENEFVMLKK

^1^ The peptide abundance weighted mean (AWM) *m*/*z* calculated for each peak group; ^2^ Heuristically ranked maximum difference, d, between the healthy epithelium, dVIN, and VSCC; ^3^ Experimental *m*/*z* of the CK5 matched nano-LC-MS/MS detected peptide; ^4^ Experimental mass of the CK5 matched nano-LC-MS/MS detected peptide; ^5^ Mascot Ions Score; ^6^ Mascot Expected Score; ^7^ CK5 matched peptide sequence.

## Supplementary Materials

Supplementary materials can be accessed at: http://www.mdpi.com/1422-0067/17/7/1088/s1.

## References

[1] Siegel R.L., Miller K.D., Jemal A. (2015). Cancer statistics, 2015. CA Cancer.

[2] Brooks L.A., Tidy J.A., Gusterson B., Hiller L., O’Nions J., Gasco M., Marin M.C., Farrell P.J., Kaelin W.G., Crook T. (2000). Preferential retention of codon 72 arginine p53 in squamous cell carcinomas of the vulva occurs in cancers positive and negative for human papillomavirus. Cancer Res..

[3] Condon J., Rumbold A., Thorn J., O’Brien M., Davy M., Zardawi I. (2009). A cluster of vulvar cancer and vulvar intraepithelial neoplasia in young australian indigenous women. Cancer Causes Control.

[4] Joura E.A. (2002). Epidemiology, diagnosis and treatment of vulvar intraepithelial neoplasia. Curr. Opin. Obstet. Gynecol..

[5] Liegl B., Regauer S. (2006). P53 immunostaining in lichen sclerosus is related to ischaemic stress and is not a marker of differentiated vulvar intraepithelial neoplasia (d-VIN). Histopathology.

[6] Van de Nieuwenhof H.P., Massuger L.F., van der Avoort I.A., Bekkers R.L., Casparie M., Abma W., van Kempen L.C., de Hullu J.A. (2009). Vulvar squamous cell carcinoma development after diagnosis of vin increases with age. Eur. J. Cancer.

[7] Van de Nieuwenhof H.P., Bulten J., Hollema H., Dommerholt R.G., Massuger L.F., van der Zee A.G., de Hullu J.A., van Kempen L.C. (2011). Differentiated vulvar intraepithelial neoplasia is often found in lesions, previously diagnosed as lichen sclerosus, which have progressed to vulvar squamous cell carcinoma. Mod. Pathol..

[8] Kokka F., Singh N., Faruqi A., Gibbon K., Rosenthal A.N. (2011). Is differentiated vulval intraepithelial neoplasia the precursor lesion of human papillomavirus-negative vulval squamous cell carcinoma?. Int. J. Gynecol. Cancer.

[9] (1990). New Nomenclature for Vulvar Disease. Report of the committee on terminology of the international society for the study of vulvar disease. J. Reprod. Med..

[10] Ridley C.M., Frankman O., Jones I.S., Pincus S.H., Wilkinson E.J., Fox H., Friedrich E.G.J.R., Kaufman R.H., Lynch P.J. (1989). New nomenclature for vulvar disease: International society for the study of vulvar disease. Hum. Pathol..

[11] Pinto A.P., Miron A., Yassin Y., Monte N., Woo T.Y., Mehra K.K., Medeiros F., Crum C.P. (2010). Differentiated vulvar intraepithelial neoplasia contains tp53 mutations and is genetically linked to vulvar squamous cell carcinoma. Mod. Pathol..

[12] Aide S., Lattario F.R., Almeida G., do Val I.C., Carvalho Mda G. (2012). Promoter hypermethylation of death-associated protein kinase and p16 genes in vulvar lichen sclerosus. J. Low. Genit. Tract Dis..

[13] Fox H., Wells M. (2003). Recent advances in the pathology of the vulva. Histopathology.

[14] Gustafsson J.O.R., Oehler M.K., Ruszkiewicz A., McColl S.R., Hoffmann P. (2011). Maldi imaging mass spectrometry (MALDI-IMS)―Application of spatial proteomics for ovarian cancer classification and diagnosis. Int. J. Mol. Sci..

[15] Balluff B., Rauser S., Meding S., Elsner M., Schöne C., Feuchtinger A., Schuhmacher C., Novotny A., Jütting U., Maccarrone G. (2011). Maldi imaging identifies prognostic seven-protein signature of novel tissue markers in intestinal-type gastric cancer. Am. J. Pathol..

[16] Marko-Varga G., Fehniger T.E., Rezeli M., Döme B., Laurell T., Végvári Á. (2011). Drug localization in different lung cancer phenotypes by maldi mass spectrometry imaging. J. Proteom..

[17] Crecelius A.C., Cornett D.S., Caprioli R.M., Williams B., Dawant B.M., Bodenheimer B. (2005). Three-dimensional visualization of protein expression in mouse brain structures using imaging mass spectrometry. J. Am. Soc. Mass Spectrom..

[18] Campello R.J., Moulavi D., Sander J. (2013). Density-based clustering based on hierarchical density estimates. Advances in Knowledge Discovery and Data Mining.

[19] Reyes M.C., Cooper K. (2014). An update on vulvar intraepithelial neoplasia: Terminology and a practical approach to diagnosis. J. Clin. Pathol..

[20] Trietsch M.D., Nooij L.S., Gaarenstroom K.N., van Poelgeest M.I. (2015). Genetic and epigenetic changes in vulvar squamous cell carcinoma and its precursor lesions: A review of the current literature. Gynecol. Oncol..

[21] Vasca V., Vasca E., Freiman P., Marian D., Luce A., Mesolella M., Caraglia M., Ricciardiello F., Duminica T. (2014). Keratin 5 expression in squamocellular carcinoma of the head and neck. Oncol. Lett..

[22] Chu P.G., Weiss L.M. (2002). Expression of cytokeratin 5/6 in epithelial neoplasms: An immunohistochemical study of 509 cases. Mod. Pathol..

[23] Khanna N., Rauh L.A., Lachiewicz M.P., Horowitz I.R. (2016). Margins for cervical and vulvar cancer. J. Surg. Oncol..

[24] Nofech-Mozes S., Khalifa M.A., Ismiil N., Saad R.S., Hanna W.M., Covens A., Ghorab Z. (2008). Immunophenotyping of serous carcinoma of the female genital tract. Mod. Pathol..

[25] Gustafsson O.J., Eddes J.S., Meding S., McColl S.R., Oehler M.K., Hoffmann P. (2013). Matrix-assisted laser desorption/ionization imaging protocol for *in situ* characterization of tryptic peptide identity and distribution in formalin-fixed tissue. Rapid Commun. Mass Spectrom..

[26] Gustafsson J.O., Oehler M.K., McColl S.R., Hoffmann P. (2010). Citric acid antigen retrieval (CAAR) for tryptic peptide imaging directly on archived formalin-fixed paraffin-embedded tissue. J. Proteom. Res..

[27] Meding S., Martin K., Gustafsson O.J., Eddes J.S., Hack S., Oehler M.K., Hoffmann P. (2012). Tryptic peptide reference data sets for maldi imaging mass spectrometry on formalin-fixed ovarian cancer tissues. J. Proteom. Res..

[28] Thiele H., Heldmann S., Trede D., Strehlow J., Wirtz S., Dreher W., Berger J., Oetjen J., Kobarg J.H., Fischer B. (2014). 2D and 3D MALDI-imaging: Conceptual strategies for visualization and data mining. Biochim. Biophys. Acta.

[29] Lokman N.A., Elder A.S., Ween M.P., Pyragius C.E., Hoffmann P., Oehler M.K., Ricciardelli C. (2013). Annexin a2 is regulated by ovarian cancer-peritoneal cell interactions and promotes metastasis. Oncotarget.

[30] Varghese F., Bukhari A.B., Malhotra R., De A. (2014). Ihc profiler: An open source plugin for the quantitative evaluation and automated scoring of immunohistochemistry images of human tissue samples. PLoS ONE.

